# Negative Mood Is Associated with Diet and Dietary Antioxidants in University Students During the Menstrual Cycle: A Cross-Sectional Study from Guangzhou, China

**DOI:** 10.3390/antiox9010023

**Published:** 2019-12-26

**Authors:** Lingling Bu, Yuting Lai, Yingyan Deng, Chenlu Xiong, Fengying Li, Li Li, Katsuhiko Suzuki, Sihui Ma, Chunhong Liu

**Affiliations:** 1 College of Food Science, South China Agricultural University, Guangzhou 510642, China; blingl@163.com (L.B.); ly0522@163.com (Y.L.); Dyinyan1@163.com (Y.D.); xchenglu1@163.com (C.X.); lfengying11@163.com (F.L.); 17671653647@163.com (L.L.); 2 The Key Laboratory of Food Quality and Safety of Guangdong Province, Guangzhou 510642, China; 3 Faculty of Sport Sciences, Waseda University, Tokorozawa 3591192, Japan; katsu.suzu@waseda.jp

**Keywords:** negative mood, diet behavior, female students, menstrual cycle, premenstrual syndrome

## Abstract

Postpubescent females may have negative mood or premenstrual syndrome during the menstrual cycle; with the emotional and physical symptoms interfering with their quality of life. Little is known about the relationship of dietary behaviors and dietary antioxidant intake with negative mood or premenstrual syndrome in university students in China; so we explored the relationship between negative mood and dietary behavior in female university students during the three menstrual cycle phases. Random sampling was used to enroll 88 individuals from a university in Guangzhou; China in the study. Data were collected using self-administered questionnaires. Descriptive statistics and multiple logistic regression analyses were performed. During the menstrual phase, tea, black coffee and carbonated beverage intake was higher in the group with a high negative affect scale score than in the low score group (*p* < 0.05). Likewise; during the premenstrual phase, fresh fruit (banana and red Chinese dates) intake was higher in the group with a high negative affect scale score than in the low-score group (*p* < 0.05). The logistic regression analysis results showed that negative mood was positively associated with tea, coffee, and carbonated beverage intake during the menstrual phase (β = 0.21, *p* = 0.0453, odds ratio = 1.23), and negative mood was positively associated with banana and red Chinese dates intake during the premenstrual phase (β = 0.59, *p* = 0.0172, odds ratio = 1.81). Our results suggest that negative mood may be associated with diet and specific food in university postpubescent females.

## 1. Introduction

Evidence indicates a higher prevalence of mental health disorders among university students than the general adult population, and that this situation is a growing area of concern. The University of Newcastle Student Healthy Lifestyle Survey 2017 revealed that 37.2% of university students had a high or very high risk of psychological distress [1]. According to the World Health Organization, mental health surveys have reported that 20–31 of university students worldwide have at least one mental health disorder [2,3].

A balanced diet plays an important role in our mental and behavioral patterns, and so our dietary patterns affect our cognition and emotions [4,5]. The ability of carbohydrate to improve mood has been frequently discussed in previous studies [6,7,8]. However, according to a meta-analysis including 31 studies and 1259 participants, acute carbohydrate intake might not have beneficial effects on any aspect of mood and might be associated with increased fatigue within 30 min after consumption [9]. In contrast, a low carbohydrate/high protein breakfast consumed over a 3-week period is also reported to cause undesired emotional fluctuations, such as increased anger [10]. In a descriptive study, participants with high soy dietary intake (>30.1 portions per month) were less prone to negative emotions than those with low or moderate soy dietary intake [11]. Overall, diet quality is frequently reported to be inversely associated with negative mood, especially in students. However, the association of specific foods with negative mood has not been extensively studied, particularly in the context of Asian dietary patterns.

Mood can influence food choice because people often eat to cope with negative feelings [12]. In a survey of 1589 college students, anxiety sensitivity was revealed to be related to eating expectancies [13]. In another emotional eating survey conducted in the United States, female undergraduates identified stress as the primary trigger for emotional eating [14]. However, given the major differences between Western and Eastern dietary patterns, questionnaires that account for local characteristics are required to correctly assess the associations between frequency of food consumption and perceived negative mood.

Female students frequently experience premenstrual syndrome (PMS) as part of their menstrual cycle [15,16]. Premenstrual syndrome is characterized by a cluster of psychological and somatic symptoms during the late luteal phase of the menstrual cycle that disappear after the onset of menses, which includes somatic symptoms, such as fatigue, appetite-change, and low energy, and affective symptoms, such as irritability, depressed mood, anxiety, and impulsive behavior. Indeed, we previously reported in a cohort study of 2579 local college students in seven cities in China that female students perceive higher stress and depression than male students according to self-reported stress scores and depression scores [17]. The influence of the menstrual cycle on the emotional state has commonly been recognized and systematically studied. 

Oxidative stress has been indicated to induce unfavorable changes in the neurotransmitter secretion network and thereby negatively affect mood [18]. Indeed, increased oxidative stress and decreased antioxidant capacity have been reported during the menstrual cycle [19]. Therefore, dietary antioxidant intake may be positively involved in the mood change during the menstrual cycle. This topic has not been extensively studied. In addition, most previous studies on emotions and diet focused on the general female populations of Western countries and few involved Asian university students. The aim of this study was to explore the associations between negative mood and dietary intake in a sample of Chinese university students. Therefore, we conducted a cross-sectional study using a Menstrual Distress Questionnaire (MDQ) to evaluate the degree of negative mood and a Food Frequency Questionnaire (FFQ) including 15 categories of foods to study the relationship between food consumption frequencies and perceived negative mood during the menstrual cycle.

## 2. Methods

### 2.1. Participants, Data Collection, and Consent

The screening conditions for participants were as follows: a normal menstrual cycle lasting between 28 and 31 days; no use of mood-regulating health products or drugs (traditional Chinese medicine or Western medicine) within the previous 3 months; and no depression or other emotional disorders. Finally, 88 female students were enrolled in the study through random cluster sampling (all were freshman, sophomore, and junior university students or postgraduates from all universities in Guangzhou city, China). Consent was obtained from all eligible students before data collection.

### 2.2. Questionnaire Distribution

The self-administered survey was distributed and collected using a sealed envelope. Participants were asked to complete the MDQ and the FFQ every night before going to bed until they had completed two menstrual cycles, including the premenstrual phase, menstrual phase, and intermenstrual phase.

### 2.3. Menstrual Distress Questionnaire

The MDQ, which was developed and improved by Moos [20] was used for emotion evaluation. It has been widely used in research into women’s emotional status during their menstrual cycle [21,22,23,24,25,26]. The MDQ comprises nine scales containing 47 items, including pain, concentration, behavioral changes, autonomic reactions, water retention, negative affect, arousal, control, and dietary habit. The negative affect scale comprises eight symptoms: crying, loneliness, anxiety, restlessness, irritability, mood swings, depression, and tension. Participants were asked to rate their level on a six-point scale ranging from no experience of the symptom to an acute or partially disabling experience of the symptom. The higher the total score of the negative affect scale, the heavier the negative mood, and vice versa. Test-retest reliabilities were performed on data from a population survey of American women and significant correlations of 0.57–0.95 were reported between symptoms in the first and second cycles [27]. A sample questionnaire is provided as Supplementary Material (Table S1).

### 2.4. Assessment of Food Frequency

Dietary behavior was assessed using the FFQ. The 15 items considered were rice, porridge, and flour products; meat and seafood; chicken eggs, duck eggs, and other eggs; vegetables; fruit; tea, coffee, and carbonated beverages; milk and dairy products; beans and bean products; sweets; puffed grains; nuts; preserves and cured meat slices; fried food; fast food; and banana and red Chinese dates. The consumption frequency of each food group in the previous week was divided into six categories: less than once a week, once a week, 2–3 times a week, 4–6 times a week, once a day, and two or more times per day. The six categories were converted into scores of 0, 1, 2.5, 5, 7, and 14, respectively, for calculating the frequencies of food consumption. A sample questionnaire is provided as Supplementary Material (Table S2).

### 2.5. Statistical Analysis

Data are presented as the mean ± standard deviation for the scale score and food consumption frequency. The participants were divided into low- and high-score groups according to the median score of the negative affect scale. Nonparametric analysis of variance was used to test for differences in the weekly frequency of food consumption between the two groups. To investigate the associations between negative affect and food consumption, multivariate logistic regression analysis was performed with categorical levels of the negative affect score as the dependent variable and food consumption frequencies as the independent variable with adjustment for age, height, and weight. Odds ratios (ORs) were calculated for the logistic regression analysis with 95% confidence intervals (95% CIs). All statistical analyses were performed using SPSS 19.0 software. A value of *p* < 0.05 was considered to be statistically significant.

## 3. Results

### 3.1. General Demographic Characteristics of the Study Population

The average age of the sample was 21.0 ± 1.6 years and the average weight was 49.5 ± 5.8 kg. Among the 88 students, 18 (20.46%) were freshmen, 29 (32.96%) were sophomores, 15 (17.05%) were junior, and 26 (29.55%) were postgraduates. The valid questionnaire rate of the survey was 98.8%.

### 3.2. Negative Affect Scale Scores

The Cronbach alpha coefficient of the negative affect scale was 0.85, with the correlation of each item ranging from 0.82 to 0.84, giving an average correlation coefficient of 0.83 (Table 1). The scores on the negative affect scale in the different phases of the menstrual cycle are also shown in Table 1. A significant difference in the negative affect scale was found between the menstrual and intermenstrual phases (*p* < 0.0001). Likewise, a significant difference was found between the menstrual and premenstrual phases (*p* < 0.0001).

### 3.3. Associations between Negative Mood and Dietary Behavior

The participants were divided into either low-score or high-score groups according to the median score on the negative affect scale (Table 2). Nonparametric ANOVA results are also shown in Table 2. During the menstrual phase, the intake of tea, black coffee, and carbonated beverages (e.g., Coke and Sprite) was higher in the high-score group than in the low-score group (*p* < 0.05). Likewise, the intake of fresh fruit (banana and red Chinese dates) was higher in the high-score group than in the low-score group during the premenstrual phase (*p* < 0.05).

The logistic regression analysis results are shown in Table 3. The results showed that negative mood was positively associated with tea, coffee, and carbonated beverage intake during the menstrual phase (β = 0.21, *p* = 0.0453, OR = 1.23) and negative mood was positively associated with banana and red Chinses dates intake during the premenstrual phase (β = 0.59, *p* = 0.0172, OR = 1.81).

## 4. Discussion

In recent years, an increasing number of women and their families have been affected more by physical and psychological irregularities related to premenstrual symptoms than by other conditions [28]. PMS, which is a constellation of physical, mood, and behavioral symptoms that are limited to the luteal phase, causes problems for many women and cannot be better explained by another diagnosis [29]. Premenstrual dysphonic disorder (PMDD) is a serious state of PMS. About 75% of women experience PMS and 14% of those with PMS have PMDD [30]. The menstrual cycle affects women’s mental health condition. A longitudinal study conducted in Australia found that women’s experience of negative mood and depressive symptoms peaks during the menopausal transition [31]. A PMS epidemiological study conducted in China showed that 33.82% of adult women have the disorder [32]. In the present study, most female students had negative mood due to their menstrual cycle. Compared with the intermenstrual or premenstrual phases, the negative mood scores were significantly higher during the menstrual phase, which is consistent with Chinese and international research results [31,32].

Social and psychological stress from daily life, abnormal endocrine secretion, and the consequences of drug consumption are all involved in the occurrence of negative mood. Excitation conduction on emotional pathways depends on neurotransmitters, such as dopamine, serotonin, and endorphin [33]. Estrogen secreted from the ovaries affects more than 400 bodily functions, including brain function via regulation of neurotransmitters [34,35]

The term estrogen encompasses three major hormones: estradiol, estriol, and estrone. Estradiol is the most potent of the three. Estrogens affect brain function, as we discussed above and as shown in numerous studies [36]. Estrogen levels vary most during the luteal phase and the fluctuating levels of estrogen secretion affect women’s mood. In a clinical trial of 15 women, the serotonin precursor was significantly increased on the days 7–11 and 17–19 of the menstrual cycle [37]. These results indicated that PMS is closely linked to mood disorders through estrogen-serotonin regulation. Molecular biology studies have shown that decreased estrogen causes the hypothalamus to release norepinephrine, thereby triggering falls in serotonin, acetylcholine, and dopamine, all of which also lead to insomnia, depression, and fatigue, which are the typical symptoms of PMS and PMDD [38].

Eating has a general effect on mood. The intake of foods, particularly calorie-dense foods such as carbonated drinks, is rewarding and pleasurable. However, our mood may be affected by the type of foods we consume. Indeed, the authors of a review article recommended the use of dietary supplements to decrease the severity of PMS symptoms [2]. Supplementation, such as conventional multivitamin supplements but particularly vitamin B6 and calcium, is reported to be negatively related to PMS-induced mood changes. Magnesium and vitamin E also help to relieve PMS symptoms. However, both carbohydrate-based drinks and long-chain free fatty acid supplementation have yielded mixed results. Total antioxidant capacity is reported to be decreased when PMS occurs, according to a randomized controlled trial conducted in 41 women in Turkey [38]. Therefore, foods rich in antioxidants might help to improve the negative mood change during PMS. Tea, coffee, and carbonated beverages contain caffeine and/or sugar. Caffeine and sugar are two of the most commonly consumed psychostimulants worldwide. They usually have positive effects on cognition, which can stimulate the central nervous system and activate the nerve center, bulbar, spinal cord center, and motor center in the cerebral cortex; they can also activate nerves, restore consciousness, dissipate fatigue, and strengthen systolic ability, making humans feel happy and even euphoric [39]. Generally speaking, both tea and coffee are rich in antioxidants such as catechins and chlorogenic acid [40]. Tea also contains theanine, which can influence a number of neurotransmitter systems. Theanine can significantly promote dopamine release and improve its physiological activity; theanine can also directly impact the concentration and activity of serotonin [41]. Serotonin and dopamine are essential for controlling behavior and emotions, and a high content of these neurotransmitters is considered to boost positive emotions because they can stimulate the nervous system, helping people to relax and feel joy [42,43]. Our study showed that negative mood was positively associated with tea, coffee, and carbonated beverage intake, with the consumption frequencies of these drinks significantly higher in the high-score group for negative mood than in the low-score group during the menstrual phase. These results indicate that university female students with a more negative mood are more likely to ingest tea, coffee, and carbonated beverages to stimulate their nervous system and alleviate their negative mood. This finding is consistent with the aforementioned research studies.

Bananas contain a large quantity of tryptophan, calcium, magnesium, and vitamin B6. Calcium is frequently used as a treatment for PMS symptoms. In a randomized controlled, double-blind crossover trial, negative affect premenstrual factors (negative affect, *p* = 0.045; water retention, p = 0.003; pain, *p* = 0.036) were significantly alleviated by calcium (1000 mg of calcium carbonate per day for 6 months) [44]. A daily supplement of 200 mg of magnesium oxide for two menstrual cycles significantly decreased PMS symptoms of weight gain, swelling of the extremities, breast tenderness, and abdominal bloating (*p* = 0.009) [45]. Tryptophan, which is the precursor to serotonin, is transformed by the enzyme tryptophan hydroxylase. Normally, tryptophan hydroxylase is not fully saturated and any increased transportation of tryptophan into the brain results in increased serotonin synthesis. Serotonin is an important neurotransmitter in the central nervous system that is involved in the modulation of various aspects of mood and behaviors, including depression, anxiety, and aggression [15]. Tryptophan from banana contributes to an increase in serotonin synthesis, with varied behavioral consequences of increased serotonergic activity. For example, higher brain levels of serotonin may improve mood in individuals. The vitamin B6 level is associated with symptoms of depression [46]. Vitamin B6 participates in the synthesis of some neurotransmitters (such as 5-hydroxytryptamine and dopamine) and plays an important role in maintaining normal mental activity. Vitamin B6 deficiency easily leads to depression [46].

Red Chinese dates contain 18 types of essential amino acids and are rich in vitamin C. Both vitamin C and vitamin B6 possess antioxidant properties. Some amino acids can regulate neurotransmitters. For instance, some amino acids related to emotions such as phenylalanine, tryptophan, tyrosine, and adenosylmethionine play important roles in combatting depression and anxiety [47]. Vitamin C is an essential nutrient required for a range of essential metabolic reactions in all animals and plants [48]. It can improve the use of iron, calcium, and folate and promote the metabolism of tryptophan and tyrosine. Hence, it is essential for neurotransmitter synthesis and will boost mood. Our study showed that negative mood was positively associated with banana and red Chinese date intake, with the banana and red Chinses date intake significantly higher in the high-score group for negative mood than in the low-score group during the premenstrual phase. This result is consistent with the aforementioned literature.

## 5. Conclusions

The current study indicates that most female students have negative mood due to the influence of the menstrual cycle and that their negative mood is related to dietary behavior. Our results suggest that negative mood may be associated with diet and specific food in university postpubescent females.

## Figures and Tables

**Table 1:** Mean scores of the negative affect scale for the menstrual, intermenstrual, and premenstrual phases and the differences among the three phases.

Phase	Mean Score	*p*
Menstrual	4.47 ± 6.70	<0.0001 (1 vs. 2)
Intermenstrual	3.21 ± 5.60	>0.05 (2 vs. 3)
Premenstrual	3.28 ± 5.42	<0.0001 (3 vs. 1)

**Table 2:** Dietary intake frequencies of the low-score and high-score groups during each menstrual phase (mean ± standard deviation).

Food Items	Menstrual Phase		Intermenstrual Phase		Premenstrual Phase	
	Low-Score Group	High-Score Group	Low-Score Group	High-Score Group	Low-Score Group	High-Score Group
Rice, porridge, and flour products	15.23 ± 3.83	16.10 ± 3.46	14.26 ± 3.49	15.76 ± 3.56	15.47 ± 3.17	15.93 ± 3.4
Meat and seafood	10.43 ± 3.21	12.34 ± 2.94	10.11 ± 2.26	12.34 ± 2.74	10.30 ± 2.20	10.85 ± 2.62
Chicken eggs, duck eggs, and other eggs	2.84 ± 1.32	3.06 ± 2.75	3.39 ± 2.77	2.88 ± 2.83	2.72 ± 2.21	3.08 ± 3.35
Vegetables	10.90 ± 3.92	10.39 ± 3.84	10.53 ± 4.24	10.93 ± 3.84	9.61 ± 4.07	10.88 ± 3.69
Fruit	4.35 ± 3.29	4.58 ± 3.28	4.41 ± 3.45	5.39 ± 3.78	4.22 ± 2.79	4.93 ± 3.25
Tea, coffee, and carbonated beverages	1.25 ± 2.07	2.31 ± 3.26 *	1.39 ± 2.06	2.36 ± 3.33	1.83 ± 2.15	1.91 ± 2.87
Milk and dairy products	3.28 ± 2.74	3.52 ± 2.93	3.14 ± 2.95	3.49 ± 2.69	3.45 ± 3.21	3.68 ± 2.78
Beans and bean products	3.08 ± 2.61	2.43 ± 2.38	2.86 ± 2.25	3.19 ± 2.91	2.78 ± 2.18	2.52 ± 2.63
Sweets	2.24 ± 2.48	2.57 ± 2.29	2.07 ± 1.40	2.43 ± 2.24	2.11 ± 2.01	2.58 ± 2.87
Puffed grains	0.80 ± 1.29	0.52 ± 0.88	0.67 ± 1.21	1.38 ± 2.41	1.09 ± 1.83	1.19 ± 1.87
Nuts	0.83 ± 1.83	1.26 ± 2.69	0.75 ± 1.37	1.30 ± 2.55	0.78 ± 1.55	1.20 ± 2.04
Preserves cured meat slices	0.99 ± 1.84	0.75 ± 1.30	0.80 ± 1.29	1.15 ± 1.83	0.60 ± 1.28	0.49 ± 0.83
Fried food	0.60 ± 0.92	0.57 ± 1.26	0.38 ± 0.57	0.48 ± 0.78	1.08 ± 2.36	0.56 ± 0.83
Fast food	0.70 ± 1.31	0.86 ± 1.20	0.82 ± 1.35	0.90 ± 1.86	0.65 ± 1.21	0.50 ± 0.88
Banana and red Chinese dates	1.24 ± 1.85	1.51 ± 2.21	1.30 ± 2.05	1.95 ± 2.41	0.68 ± 0.96	1.91 ± 2.44 *

*: *p* < 0.05.

**Table 3:** Logistic regression analysis between negative affect and food consumption frequencies.

Food Items	Menstrual Phase			Intermenstrual Phase			Premenstrual Phase		
	OR ^*^	95% CI	*p*	OR ^*^	95% CI	*p*	OR ^*^	95% CI	*p*
Rice, porridge, and flour products	1.00	0.89–1.13	0.9418	1.10	0.96–1.26	0.1551	0.98	0.88–1.09	0.6811
Meat and seafood	1.01	0.96–1.15	0.2797	1.09	0.97–1.23	0.1643	1.03	0.93–1.15	0.5437
Chicken eggs, duck eggs, and other eggs	0.99	0.81–1.22	0.933	0.82	0.66–1.00	0.0542	0.96	0.76–1.22	0.7591
Vegetables	1.00	0.86–1.17	0.9871	0.97	0.84–1.13	0.7184	1.01	0.88–1.17	0.8422
Fruit	1.00	0.83–1.20	0.9744	1.07	0.91–1.26	0.4078	0.98	0.79–1.22	0.8729
Tea, coffee, and carbonated beverages	1.23	1.00–1.51	0.0453 *	1.16	0.92–1.46	0.206	1.04	0.85–1.26	0.7347
Milk and dairy products	1.04	0.86–1.26	0.6965	1.04	0.85–1.28	0.6744	0.97	0.80–1.17	0.7326
Beans and bean products	0.89	0.71–1.12	0.3272	0.91	0.68–1.21	0.4943	0.79	0.60–1.06	0.1175
Sweets	1.09	0.87–1.37	0.4498	1.12	0.81–1.56	0.4894	1.07	0.82–1.39	0.609
Puffed grains	0.53	0.27–1.03	0.0591	1.37	0.93–2.02	0.1114	1.29	0.89–1.86	0.1752
Nuts	1.19	0.88–1.60	0.2528	1.11	0.84–1.45	0.4671	1.29	0.94–1.77	0.1131
Preserves cured meat slice	0.89	0.61–1.28	0.5221	1.03	0.70–1.50	0.8844	0.86	0.50–1.46	0.5681
Fried food	0.96	0.56–1.64	0.8681	1.17	0.47–2.90	0.7242	0.66	0.37–1.19	0.1657
Fast food	1.23	0.78–1.93	0.3813	0.89	0.60–1.28	0.5004	0.82	0.50–1.36	0.4475
Banana and red Chinese dates	1.06	0.78–1.44	0.7257	1.02	0.79–1.33	0.8738	1.81	1.11–2.96	0.0172 *

*: *p* < 0.05; OR = odds ratio, CI = confidence interval.

## Supplementary Materials

The following are available online at https://www.mdpi.com/2076-3921/9/1/23/s1, Table S1: Menstrual Distress Questionnaire, Table S2: Food Frequency Questionnaire.
