# Supplementary material for: Negative Mood Is Associated with Diet and Dietary Antioxidants in University Students During the Menstrual Cycle: A Cross-Sectional Study from Guangzhou, China
Source: Antioxidants (Basel). 2019 Dec 26;9(1):23. doi: 10.3390/antiox9010023 (PMC7023165; doi:10.3390/antiox9010023)
Supplement: Supplementary file 1 [file antioxidants-09-00023-s001.zip › supplementary/Supplementary Table 1.pdf]

Supplementary Table 1

ID:

Menstrual Distress Questionnaire (MDQ)

Basic information

Date:

Is today the first day or last day of your menstrual period?

Today is the first day of my menstrual period ()

Today is the last day of my menstrual period ()

Today is neither the first day nor the last day of my menstrual period.

Symptom Scale

This questionnaire contains 47 items. The score of each item ranges from "0" to "6", from "no such experience or symptom" to "this experience or symptom has seriously affected daily life". If the score is 0 points, you can leave this item blank, and the rest will be scored according to the actual situation.

Pain
- Headache
- Cramps
- Backache
- Chest pain
- Breast pain
- Prickling or stinging sensation
- I have other pains

Concentration
- Difficulty concentrating
- Accidents
- Confusion

Behavioral Change
- Take naps; stay in bed
- Stay at home
- Changes in eating habits
- Decreased efficiency
- Avoid social activities

Autonomic Reaction
- Dizziness, faintness
- Nausea, vomiting
- Muscle stiffness
- Insomnia

Water Retention
- Weight gain
- Painful breasts
- Swelling

- Feeling hot
- Skin deterioration

Negative Affect

- Irritability
- Mood swings
- Depression
- Crying
- Tension
- Anxiety
- Weariness
- Loneliness
- Bad memory
- Decreased judgment
- Study or work performance decline
- Decrease in efficiency

Arousal

- Well-being
- Energy, activity
- Calm
- Joy
- Peace

Control

- Restrained
- Ringing in ears
- Poor eyesight
- Cold sweats
- Rapid heartbeat
- Reduced motor coordination
