# Supplementary material for: Negative Mood Is Associated with Diet and Dietary Antioxidants in University Students During the Menstrual Cycle: A Cross-Sectional Study from Guangzhou, China
Source: Antioxidants (Basel). 2019 Dec 26;9(1):23. doi: 10.3390/antiox9010023 (PMC7023165; doi:10.3390/antiox9010023)
Supplement: Supplementary file 1 [file antioxidants-09-00023-s001.zip › supplementary/Supplementary Table 2.pdf]

Supplementary Table 2

ID: Date:

Height: Weight: Age: Grade:

Food frequency questionnaire

For each food listed, fill in the circle indicating your average total consumption of the amount specified during the past week.

① Less than once per week ② Once per week ③ 2–3 times per week

④ 4–6 times per week ⑤ Once per day ⑥ More than once per day

1. Staple foods
A. Rice and porridge ()
B. Flour products (noodles, buns, steamed buns) ()

2. Eggs, meat, and poultry
C. Red Meat (pork, lamb, beef) ()
D. Poultry (chicken, duck, goose) ()
C. Seafood (fish, shrimp, crab) ()
E. Eggs ()

3. Fruit, vegetables, and nuts
F. Fresh vegetables ()
G. Fresh fruit and fresh juice ()
H. Nuts ()

4. Beverages
I. Tea, coffee, or carbonated beverages [[In the main text, you say "carbonated beverages".]]

5. Dairy and soybean
J. Dairy (milk or skimmed milk, cheese, yoghurt) ()
K. Soybean products ()

6. Processed foods
L. Sweet foods (pastries, chocolate, ice cream, waffles, cakes, candies) ()
M. Puffed grains ()
N. Dried fruit or cured meat ()
O. Fried foods ()
P. Fast food ()

8. Special foods
Q. Spirulina products ()
R. Protein powder ()
S. Melatonin products ()

T. Bananas and red Chinese dates ()
